# Drug testing complementary metal‐oxide‐semiconductor chip reveals drug modulation of transmitter release for potential therapeutic applications

**DOI:** 10.1111/jnc.14815

**Published:** 2019-07-31

**Authors:** Meng Huang, Shailendra S. Rathore, Manfred Lindau

**Affiliations:** ^1^ Department of Materials Science and Engineering Cornell University Ithaca New York USA; ^2^ School of Applied and Engineering Physics Cornell University Ithaca New York USA

**Keywords:** amperometry, bupropion, citalopram, CMOS, patch clamp

## Abstract

Neurodegenerative diseases, such as Parkinson’s disease, Alzheimer’s disease, and Huntington’s disease, are considered incurable and significantly reduce the quality of life of the patients. A variety of drugs that modulate neurotransmitter levels have been used for the treatment of the neurodegenerative diseases but with limited efficacy. In this work, an amperometric complementary metal‐oxide‐semiconductor (CMOS) chip is used for high‐throughput drug testing with respect to the modulation of transmitter release from single vesicles using chromaffin cells prepared from bovine adrenal glands as a model system. Single chromaffin cell amperometry was performed with high efficiency on the surface‐modified CMOS chip and follow‐up whole‐cell patch‐clamp experiments were performed to determine the readily releasable pool sizes. We show that the antidepressant drug bupropion significantly increases the amount of neurotransmitter released in individual quantal release events. The antidepressant drug citalopram accelerates rapid neurotransmitter release following stimulation and follow‐up patch‐clamp experiments reveal that this is because of the increase in the pool of readily releasable vesicles. These results shed light on the mechanisms by which bupropion and citalopram may be potentially effective in the treatment of neurodegenerative diseases. These results demonstrate that the CMOS amperometry chip technology is an excellent tool for drug testing to determine the specific mechanisms by which they modulate neurotransmitter release.

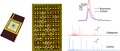

## Graphical abstract

Bupropion and citalopram are antidepressant drugs that have been used for decades. However, the effect of these two drugs on the release frequency, quantal size, and kinetics of neurotransmitter release events has not been studied before. In this work, we used a surface‐modified complementary metal‐oxide‐semiconductor integrated circuit sensor array to perform high‐throughput amperometry recordings of chromaffin cells treated with these two drugs. The results show that bupropion increases the quantal size of neurotransmitter release events and citalopram increases the release frequency by increasing the readily releasable pool sizes for chromaffin cells. The potential therapeutic applications of the two drugs on neurodegenerative diseases have been discussed.

**Abbreviations used**
AD: Alzheimer’s disease
BSA: bovine serum albumin
CMOS: complementary metal‐oxide‐semiconductor
DA: dopamine
HD: Huntington’s disease
IC: integrated circuit
NE: norepinephrine
PD: Parkinson’s disease
RRP: readily release pool
SEM: standard error of the mean
SSRI: selective serotonin reuptake inhibitor
VMAT: vesicular monoamine transport

With increasing human life expectancy, the incidence of neurodegenerative diseases has been increasing over the decades and yet the mechanisms behind these diseases are still not fully elucidated (Martorana *et al.* 2010; Batista and Pereira 2016). For the treatment of these diseases, drugs that modulate transmitter release play an increasingly significant role. Thus, techniques for high‐throughput testing and characterization of drugs that may potentially modulate transmitter release are urgently needed. The most commonly used treatment for Parkinson’s disease (PD), l‐Dopa, increases the quantal size of transmitter release events (Pothos *et al.* 1996; Gong *et al.* 2003). Cognitive decline in Alzheimer’s disease (AD) is associated with the interplay among different neurotransmitters, including dopamine, serotonin, noradrenaline, or glutamate (Martorana *et al.* 2010). Among the symptoms in PD, AD, and Huntington’s Disease (HD), depression is commonly found accompanying these diseases (Grimes *et al.* 2012; Beglinger *et al.* 2014; Cooney and Stacy 2016; Aboukarr and Giudice 2018), making the treatment more complex.

Bupropion and citalopram are antidepressant drugs. Ever since its first introduction in the 1980s, bupropion has been widely used as a prescription drug for depression treatment. However, its mechanism of action remains unclear (Dwoskin *et al.* 2006; Paterson 2009). Bupropion is a dopamine and norepinephrine reuptake inhibitor (Ferris and Beaman 1983; Ascher *et al.* 1995; Damaj *et al.* 2004), a vesicular monoamine transport enhancer for cytoplasmic dopamine to accumulate in vesicles (Rau *et al.* 2005) and an anti‐inflammatory drug (Kast 2003). It has been reported that bupropion is also an effective drug for the treatment of PD for some patients (Cooney and Stacy 2016). Citalopram is considered a selective serotonin reuptake inhibitor (Sindrup *et al.* 1992; Tseng *et al.* 2010; Cadeddu *et al.* 2014) and restores short‐term memory deficits in AD patients (Zhang *et al.* 2018). While a few studies reported modulation of transmitter release in synaptosomes and in rat brain by bupropion or citalopram (Lin *et al.* 2011; Cadeddu *et al.* 2014), there are no studies investigating how bupropion and citalopram modulate frequency, quantal size, and kinetics of individual transmitter release events on a single‐cell, single event level, and the relation of these mechanisms with the efficacy of neurodegenerative disease treatment.

Amperometry is a powerful method to characterize quantal transmitter release events measuring the kinetics, quantal size, and fusion pore properties of exocytosis in exquisite detail. In amperometry, neurotransmitters released from the cells, such as dopamine, norepinephrine, and epinephrine, are oxidized at the electrode which is typically set at 700 mV versus a Ag|AgCl electrode, generating an oxidation current which reflects the quantal size and kinetics of the vesicle release event. Recent advances on complementary metal‐oxide‐semiconductor (CMOS) technology have provided technology for development of CMOS‐based biosensors, including devices specifically designed for amperometry measurements (Ayers *et al.* 2007; Ayers *et al.* 2010; Kim *et al.* 2013; Ghoreishizadeh *et al.* 2017; Dorta‐Quinones *et al.* 2018). CMOS technology features highly customizable and scalable electronic chip design, which enables the integration of several electronic components such as electrodes and amplifiers on one chip. These devices typically feature 100‐1000 working electrodes on a small ~ 15 mm^2^ silicon substrate with potentiostats and amplifiers integrated on chip. SU‐8 microwell structures have also been incorporated on chip for reliable, precise, and highly parallel single‐cell trapping at individual electrodes, facilitating the measurement efficiency (Huang *et al.* 2018). Here, we employed surface‐modified CMOS devices with integrated microwells for amperometric parallel recordings to measure simultaneously the effect of bupropion or citalopram on the quantal size, kinetics, and frequency of transmitter release on multiple single cells on the single vesicle level. The aim of this study is to relate the therapeutic potential of bupropion and citalopram to a mechanistic understanding of the modulation of the quantal release process by these drugs.

## Materials and methods

### Ethical and experimental information

Institutional ethical approval was not required for this study. This work was not pre‐registered. No blinding procedures were performed.

### Buffer solutions

Standard extracellular buffer contained (in mM) 140 NaCl (CHEBI: 26710, cat no.: 7581‐06, Macron Fine Chemicals), 5 KCl (CHEBI: 32588, cat no.:6858‐04, Mallinckrodt Chemicals, St Louis, MO, USA), 1 MgCl_2_ (CHEBI: 6636, cat no.: AM9530G, Invitrogen by Thermo Fisher Scientific, Waltham, MA, USA), and 10 HEPES (CHEBI: 46756, cat no.: 15630080, Gibco, Grand Island, NY, USA)/NaOH (CHEBI: 32145, cat no.: 72074, Fluka Chemika, Buchs, Switzerland) with pH adjusted to 7.3 and the osmolarity adjusted to 315 mmol/kg with glucose (CHEBI: 42758, cat no.: D16‐500, Fisher Scientific, Fair Lawn, NJ, USA). The high K^+^ stimulation buffer contained (in mM) 105 KCl, 5 CaCl_2_ (CHEBI: 3312, cat no.: 21115, Sigma‐Aldrich, St Louis, MO, USA), 0.7 MgCl_2_, and 10 HEPES/NaOH with pH adjusted to 7.3. FFN511 (cat no.: ab120331, Abcam, Cambridge, UK, 2017) was dissolved in dimethyl sulfoxide to make 20 mM stock solutions. Shortly before the experiment, FFN511 solution was diluted with standard buffer with a final concentration of 7 μM. The FFN511 containing standard buffer was further adjusted with an osmolarity of 315 mmol/kg. Bupropion hydrochloride (5 μM; CHEBI: 3220, cat no.: B102, Sigma‐Aldrich, 2017) was added to the standard extracellular buffer and the high K^+^ stimulation buffer, respectively. The resulting bupropion containing standard buffer and stimulation buffer were further adjusted to an osmolarity of 315 mmol/kg. Citalopram hydrobromide (CHEBI: 3724, cat no.: C7861, Sigma‐Aldrich, 2017) was added to the standard extracellular buffer and the high K^+^ stimulation buffer at a final concentration of 1 μM, respectively. The resulting citalopram containing buffers were further adjusted to an osmolarity of 315 mmol/kg. The high K^+^ stimulation buffer used in bupropion and citalopram experiments contained the same concentration of bupropion and citalopram with the corresponding standard buffer to maintain the same drug levels in the cells during the recordings after stimulation. For barium experiments, calcium was replaced by 5 mM BaCl_2_ (CHEBI: 86153, cat no.: 11760, Fluka Chemika) in the extracellular buffer for stimulation of the cells.

The cell preparation buffer contained (in mM) 118 NaCl, 3.3 KCl, 1 NaH_2_PO_4._H_2_O (CHEBI: 37585, cat no.: 7892, Mallinckrodt Chemicals), 1 MgSO_4_.7H_2_O (CHEBI:32599, cat no.: M63‐500, Fisher Scientific), 10 glucose, 25 HEPES‐NaOH, 2 l‐Glutamine (CHEBI: 18050, cat no.: 25030‐081, Gibco), 100 units‐mg/mL Pen‐Strep (cat no.: 15140‐122, Gibco), 0.1 mg/mL Gentamycin (CHEBI: 102135, cat no.: 15750‐060, Gibco), supplemented with MEM vitamins (cat no.: 11120‐052, Gibco) and MEM amino acids (cat no.: 11140‐050, Gibco) according to manufacturer’s instructions. Collagenase buffer is made by adding 1 mg/mL collagenase (CHEBI: 3823, cat no.: C0130, Sigma‐Aldrich) to the cell preparation buffer with bovine serum albumin (BSA, cat no.: A7906, Sigma‐Aldrich).

The culture media contained (in mL) 200 Dulbecco’s modified eagle medium/nutrient mixture F‐12 (cat no.: 10565‐018, Gibco), 200 Ham’s nutrient mixture F‐12 (cat no.: 11765‐054, Gibco), 40 fetal bovine serum (cat no.: 16000‐044, Gibco), 4 Pen/strep, 4 l‐Glutamine (0.2M), 9 HEPES/NaOH (1M), 4.6 insulin‐transferrin‐selenium‐ethanolamine (100X, cat no.: 51500‐056, Gibco). All custom‐made materials will be shared upon reasonable request.

### Amperometry sensor array fabrication

The CMOS sensor array integrated circuit devices with SU‐8 trapping wells (RRID: SCR_017082) were fabricated and prepared as previously described (Kim *et al.* 2013; Huang *et al.* 2018). After post‐fabrication, the devices were conditioned by culturing live chromaffin cells on the devices for a week, followed by cleaning with water. The devices were further conditioned by culturing live chromaffin cells for a second week. For re‐use of the devices after each measurement, the devices were cleaned with strong stream of water.

### Cell preparation and culture on the device

Two bovine adrenal glands were provided by a local slaughter house (Owasco Meat Co., Inc., Moravia, NY, USA) and used for each preparation of chromaffin cells to achieve the maximum available number of cells. Bovine chromaffin cells were prepared as previously described (Parsons *et al.* 1995). The bovine chromaffin cell preparations were performed in accordance with an institution approved protocol (1999‐0015). Briefly, the glands were cleaned and perfused through the aortal opening by connecting to a perfusion setup having carbogen flow of 3–4 bubbles per second all the time. The perfusate was allowed to drain through the several cuts made at the distal end of the gland for 1 h at 37°C. Then, the glands were digested by another perfusion with collagenase buffer for 1 h at 37°C, followed by being cut and opened in a sterile dish kept inside a laminar flow hood to collect the medulla in smallest possible clumps. All the clumps were gently homogenized and re‐incubated with collagenase buffer in a sterile closed flask vented for carbogen flow. After 1 hr of incubation at 37°C, cells were filtered and washed twice with cell preparation buffer with BSA by centrifugation at 500 rpm for 10 min. Cell pellet was resuspended in culture medium and filtered before plating onto the device.

Four cell preparations were used for bupropion experiments and three for citalopram experiments. Two cell preparations were used for citalopram barium experiments. Cell suspension solution was plated directly on a chip for same day measurements. Twenty microliters of media was plated on the device and placed in the incubator at 37ºC and 8% CO_2_ for 4 h. Another 20 μL of cell suspension in media with 12 000 cells was then added on the device and cultured in the incubator at 37ºC and 8% CO_2_ for 2 h.

Before amperometric on‐chip recording for the control group, the culture media on the devices was exchanged with 40 μL of standard extracellular solution. To stimulate all the cells on the chip simultaneously, stimulation was performed by addition of 80 μL of the 105 mM K^+^ stimulation buffer to the 40 μL volume of standard extracellular solution already present, resulting in a final K^+^ concentration of 70 mM. For drug treatments, the culture media on the devices was exchanged with 80 μL of buffer containing either 5 μM bupropion or 1 μM citalopram and incubated for 15 min or 30 min. For stimulation, 40 μL of drug containing buffer was removed and 80 μL of the bupropion or citalopram containing high K^+^ stimulation buffer added, providing a final K^+^ concentration of 70 mM.

FFN 511 on chip fluorescence imaging was performed with a Zeiss Axioskop 2 FS plus microscope with a Zeiss Achrostigmat 5×/0.12 objective lens. Fluorescence excitation was generated by a mercury arc lamp (HBO 100) through a 395–440 nm excitation filter and a 460 nm dichroic. The emission fluorescence passed through a 535‐nm emission filter and the images were acquired with a CMOS camera (AmScope MU 500).

### Sensor array recording, data acquisition, and amperometric spike analysis

The experimental set up and data acquisition were previously described (Huang *et al.* 2018). The set up includes a custom‐assembled breadboard which provides the necessary electrical connection for the CMOS chip, two DC power sources (36311A, Keysight Technologies, CA, USA), and a multifunction data acquisition module (PXIe‐6368, National Instrument, TX, USA). Data acquisition was performed using the NIDAQ Tools MX interface based on Igor Pro 6.2. The sampling rate for all experiments was 2 kS/s. Amperometry data analysis for live‐cell recordings was conducted with IGOR‐based Quantal Analysis software (Mosharov and Sulzer 2005). The minimum amperometric spike amplitude was set to 0.5 pA. Quantal size, maximum current, half width, parameters for the rising phase, foot current, foot duration, and foot quantal size were used to compare between the treated and the control groups. The foot amplitude is determined as the ratio of the foot quantal size and the foot width.

### Whole‐Cell patch‐clamp recording

Standard whole‐cell patch‐clamp recordings were performed as previously described (Zhao *et al.* 2016) to measure capacitance changes and Ca^2+^ currents and determine readily release pool (RRP) size. Patch pipettes were pulled to have resistances of around 3 MΩ with intracellular solutions. Data were recorded with an EPC‐9 amplifier and sampled at 10 kHz with Patch Master (HEKA, Holliston, MA, USA). Data analysis was performed with customized IGOR Pro programs. The pipette intracellular solution contained (in mM) 145 Cs‐glutamate, 8 NaCl, 1 MgCl_2_, 0.18 CaCl_2_, 0.25 BAPTA, 10 HEPES‐CsOH, 2 Mg‐ATP, and 0.5 Na‐GTP. The patch extracellular solution contained (in mM) 145 NaCl, 2.8 KCl, 1 MgCl_2_, 5 CaCl_2_, 10 HEPES‐NaOH, 10 glucose supplemented with 5 μM bupropion or 1 μM citalopram. The solutions were adjusted to a pH of 7.3 and an osmolarity of 315 mmol/kg. Cells were prepared as described above and cultured in glass‐bottom culture dishes (MatTek, Ashland, MA, USA). Before the patch‐clamp experiment, the culture media was exchanged with the patch extracellular solution containing bupropion or citalopram, and cells were further incubated for 15 min or 30 min at 22–24°C, respectively. Cells in the control group were used for measurement immediately after exchanging the culture media with the patch extracellular solution. The pulse protocol of the patch‐clamp recordings included five individual depolarizing pulses from −70 to + 10 mV with 100 ms pulse widths. The first two pulses separated by 200 ms were used to determine the RRP sizes from the capacitance changes. The third pulse given 1 s after the second pulse was used to assess depletion of the RRP. Ten seconds after the third pulse, a fourth and a fifth pulse were given, also separated by 200 ms to determine the level of refilling of the RRP. Each cell was measured twice with the same pulse protocol with a 2‐minute rest time between the two measurements. Ca^2+^ entry was quantified as the integral of the calcium current during the 100 ms pulses. If the cell broke or the GΩ seal was lost during the measurement, the corresponding data were excluded from the analysis. Three cell preparations were used for bupropion and citalopram patch experiment.

### Statistical analysis

**Figure 1:**
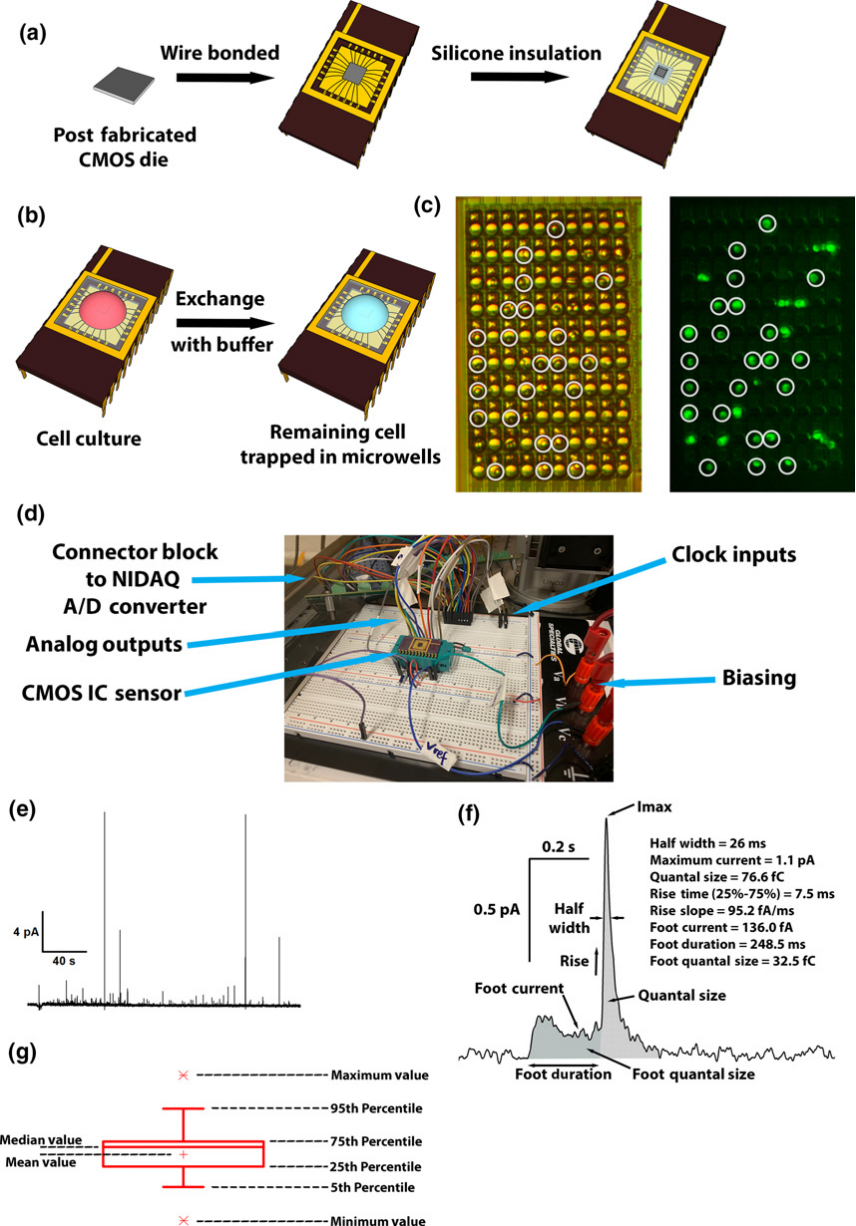
(a) Complementary metal‐oxide‐semiconductor devices were post‐fabricated with shifted electrodes and SU‐8 microwell structures, followed by packaging and insulation with silicone. (b) Cells were cultured on chip and culture media was exchanged with standard buffer after settling down. Remaining cells were trapped inside the microwells. (c) Bright field and fluorescent images showing chromaffin cells labeled with FFN 511 trapped in the SU‐8 microwells on the chip surface. Solid white circles indicate cells trapped individually in the microwells. Other microwells with fluorescence trapped more than one cell inside, and were therefore not included in the data analysis. (d) Complementary metal‐oxide‐semiconductor integrated circuit chip was plugged into self‐assembled data acquisition device for amperometry recordings. (e) An example amperometric trace recorded by one of the electrodes. (f) An example amperometric spike on expanded scale showing the analyzed amperometric spike parameters. The area in gray indicates the foot quantal size and the area with diagonal line pattern indicates the quantal size of the entire release event. (g) Description of all information shown in the box plots.

All amperometric parameters are presented in mean ± standard error of the mean (SEM). Patch‐clamp experiment data are shown in box plot and a detailed description of all symbols is provided in Fig. 1(g). Kolmogorov–Smirnov test was performed to determine the normality of the data. For normal distributed data, *P* values and significance were determined by one‐way anova. For non‐normal distributed data, *P* values and significance were determined by Kruskal–Wallis one‐way anova. All *P* values are listed in Table S1. Statistical analysis and drawings were performed in Igor Pro 6.2 and OriginPro 2015. No predetermination of the sample size was performed before the experiment. No test of outliers was conducted. To achieve the statistical significance, amperometry data were collected through multiple experiments with multiple cell preparations. Median values of the amperometry parameters were taken from all events of each individual cell. The median parameters from the two groups were averaged and compared only when they were from the same preparation. The final parameters were normalized to the data from the control group of the first preparation.

## Results

CMOS devices were post‐fabricated with shifted electrodes and SU‐8 microwells (Huang *et al.* 2018), and packaged for recordings, as shown in Fig. 1(a). To trap cells individually in these microwells, chromaffin cells were cultured on chip and the cell culture media was exchanged with standard buffer after the cells had settled in the microwells (Fig. 1b and c). Cells trapped inside the microwells remained on chip after the standard buffer wash. Figure 1(c) shows a bright field and a fluorescence image of trapped chromaffin cells labeled with the false fluorescent neurotransmitter (FFN) 511 in the SU‐8 microwells. White circles indicate cells trapped individually inside these microwells. Fluorescent spots without circle were either not inside a microwell or consisted of two or more cells. These were not included in the analysis. Following acquisition of the microscope images, the CMOS IC sensor was plugged into a self‐assembled data acquisition device for amperometry recordings (Fig. 1d) and the cells were stimulated by application of high [K^+^]. A typical amperometric trace recorded by one of the chip electrodes is presented in Fig. 1(e). The analyzed amperometry parameters are indicated in the example spike in Fig. 1(f). Quantal size, maximum current, half width, parameters for the rising phase, foot current, foot width, and foot quantal size were quantified and compared between the treated and the control groups. The foot amplitude is determined as the ratio of the foot quantal size and the foot width. Changes in foot current preceding the amperometric spike reflect changes in the properties of the initial fusion pore, which is important for the efficiency of synaptic transmission. To test the statistical significance, data were collected through multiple experiments with multiple cell preparations. The parameters from the control group and the drug‐treated group were averaged and compared among cell populations from the same preparation. To pool data from multiple preparations, the parameters were normalized to the data from the control group of the respective preparation.

### Bupropion increases quantal size of individual release events

**Figure 2:**
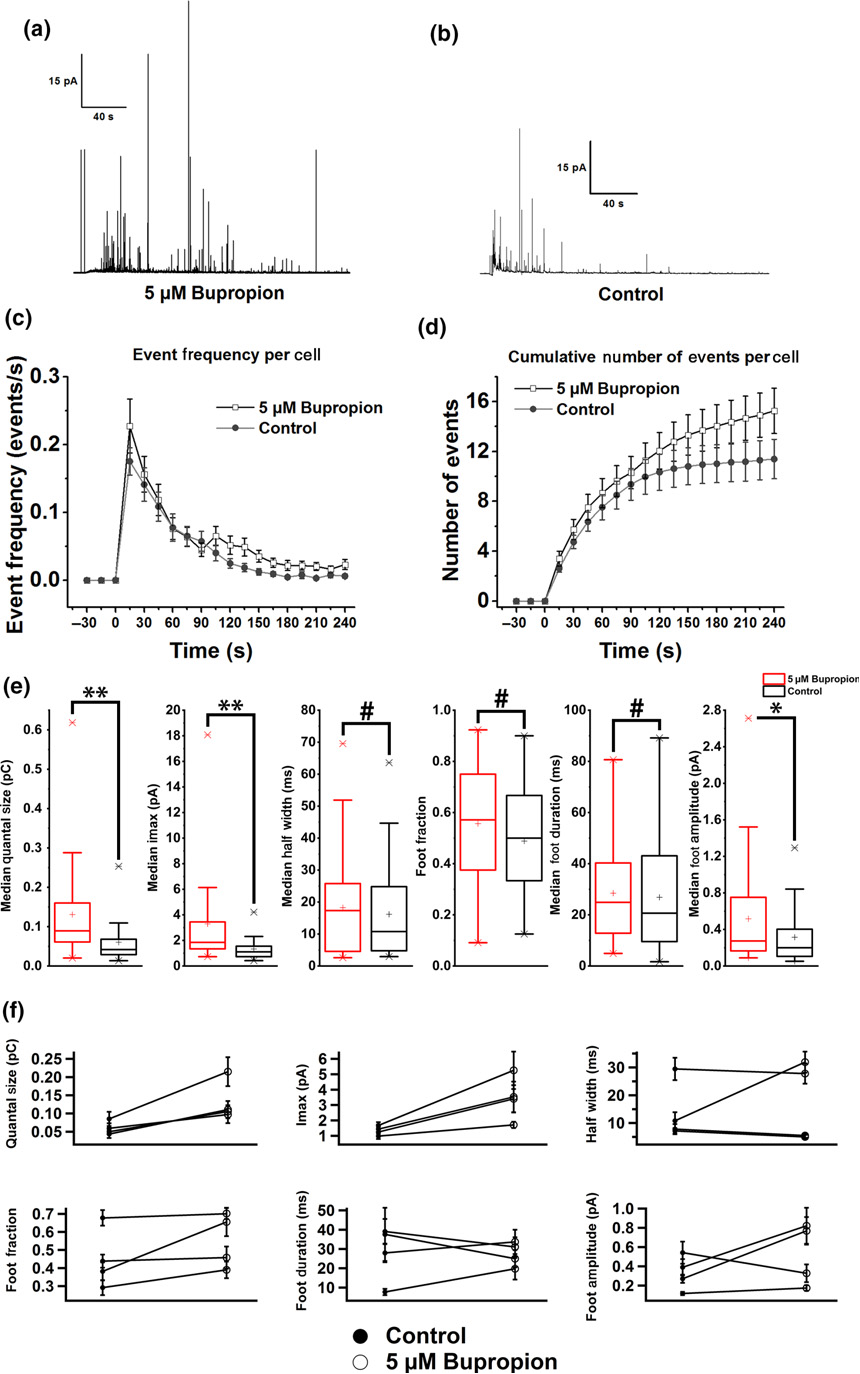
Modulation of quantal transmitter release events by bupropion. (a) Example amperometry traces for a bupropion‐treated cell and (b) a cell in the control group. (c) Average event frequency per cell during the measurement and (d) cumulative number of events per cell during the entire recording. The differences were not significant for both (c) and (d). (e) Box plots of median amperometry parameters for cells treated with 5 μM bupropion (red) and the control group (black). Differences were statistically significant for quantal size, maximum current, and foot amplitude. (f) Scatter plots for all amperometry parameters. Each data point represents the mean of medians of all cells from the same preparation. All error bars are in SEM. *p < 0.05, **p < 0.01, ^#^ *p *> 0.05. For control group, *n* = 43 cells, for bupropion‐treated group, *n* = 49 cells, 4 preparations.

Amperometry data were collected from 49 bupropion (5 μM)‐treated cells and 43 cells in the control group. Figure 2(a) and (b) show two example traces for the bupropion‐treated group and the control group, respectively. Figure 2(c) and (d) show the event frequencies for the two groups of cells. No significant differences were found for the event frequencies during the first 90 s between the bupropion‐treated group and the control group. After 90 s, the bupropion‐treated group shows a slightly higher event frequency than the control group, but the difference was not significant with *p *> 0.05.

Comparison of the normalized amperometric spike parameters from cells treated with bupropion with those from the control group revealed that bupropion significantly increased quantal size and maximum current spike amplitude (Fig. 2e and f). The median quantal size was boosted by 120%, while the median maximum current was potentiated by 151%. The median foot amplitude was also enhanced by 76% while the kinetic parameters (half width, foot duration, and fraction of spikes showing a foot signal) were unchanged (Fig. 2e and f). These results indicate that individual vesicles release more than twice the number of transmitter molecules after bupropion treatment but the release kinetics are unchanged.

No significant difference was found between bupropion‐treated and control cells for the kinetics of individual release events quantified by the mean of the median half width determined for each cell (Fig. 2e and f). To determine if bupropion treatment affects the kinetics of release events differently depending on quantal size, a box analysis was performed (Machado *et al.* 2002), which showed that the release kinetics from larger vesicles were more susceptible to bupropion while small vesicles tended to be less affected (Fig. S1).

It has been shown that the quantal size recorded from a variety of cells does not fit a Gaussian distribution (Finnegan *et al.* 1996). Interestingly, the cube roots of the quantal sizes show a better fit with Gaussian distribution because the vesicle radii are normally distributed (Bekkers *et al.* 1990). Figure S2(a) and (b) shows the double Gaussian fit and the cumulative double Gaussian fit for the histogram and the cumulative histogram of the cube roots of the quantal sizes, respectively. Both fits align well with the histograms and yield very similar parameters, indicating the existence of two size populations of vesicles, as previously reported for mouse chromaffin cells (Grabner *et al.* 2005). The cumulative double Gaussian fit was used for all other cells because of the smaller errors. The ratio of the mean values of the two vesicle populations (Fig. S2c) and the weight of the larger vesicle population (Fig. S2d) are similar between the bupropion‐treated group and the control group. Bupropion equally potentiates both vesicle populations to larger quantal sizes.

### Citalopram increases the readily releasable pool of vesicles

**Figure 3:**
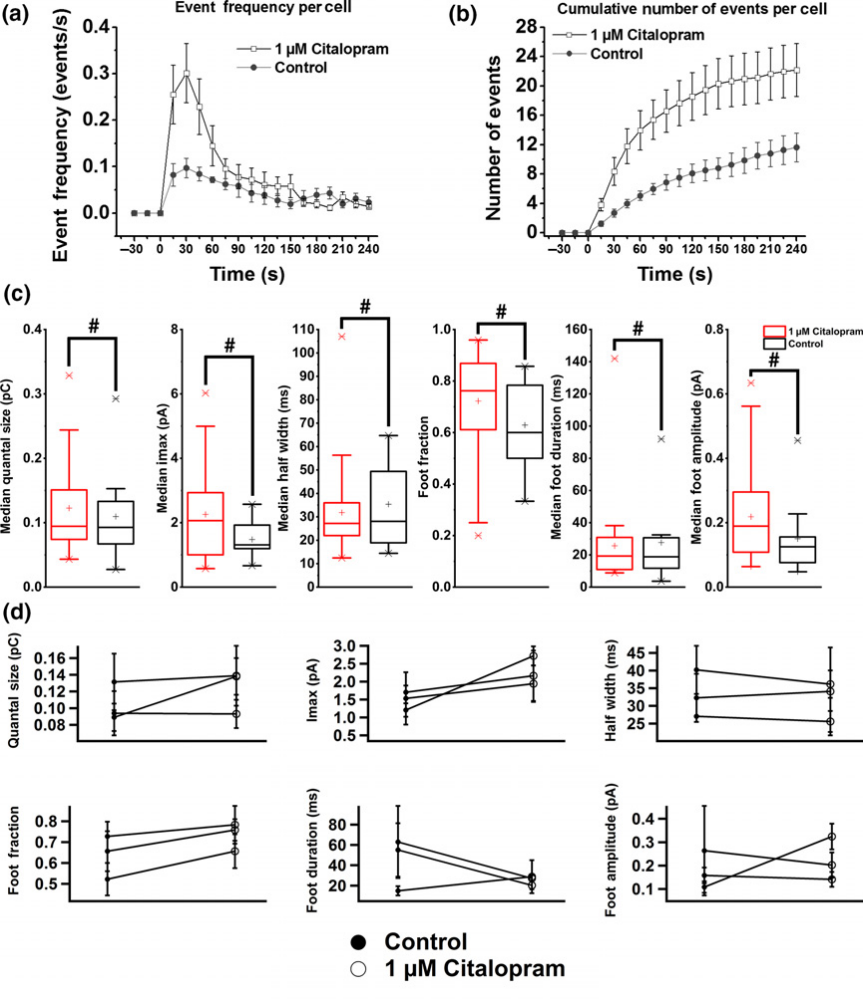
(a) Average event frequency per cell during the measurement and (b) cumulative number of events per cell during the entire recording with error bar in SEM. The differences were significant with *p* < 0.05 and *p* < 0.01 for the event frequency for the first 60 s and cumulative number of events, respectively. After stimulation, the release frequency was greatly increased for the first 60 s. (c) Box plots of median amperometry parameters for cells treated with 1 μM citalopram (red) and the control group (black) with error bar in SEM. No significant differences were observed. (d) Scatter plots for all amperometry parameters. Each data point represents the mean of medians of all cells from the same preparation. ^#^ *p *> 0.05. For control group, *n* = 17 cells, for citalopram‐treated group, *n* = 23 cells, 3 preparations.

To determine the mechanism by which citalopram modulates transmitter release, quantal release events from 23 cells treated with 1 μM citalopram and 17 cells in the control group were measured and analyzed. Citalopram‐treated cells showed a large increase in initial release frequencies compared to the control group, (Fig. 3a and b). During the first 45 s, the release frequency for the citalopram‐treated cells was about three times higher than that in control cells and gradually decreased afterwards. After 90 s, the release frequencies for both citalopram‐treated cells and the control group were very similar. In contrast to bupropion, the amperometric spike parameters for cells treated with 1 μM citalopram showed no statistically significant difference compared to control cells (Fig. 3c and d). The median maximum current and the foot amplitude were slightly increased in citalopram‐treated cells but, the differences did not reach significance.

A possible explanation for the increase in the initial release frequency in the citalopram‐treated cells (Fig. 3a and b) may be an increased readily releasable pool (RRP) of secretory vesicles. It has been shown that barium stimulation of chromaffin cells evokes release from vesicles in the reserve pool but not from the RRP (Duncan *et al.* 2003; Wiegand *et al.* 2003; Haynes *et al.* 2007). If citalopram selectively increases the RRP, it should thus not affect the release frequency in response to barium stimulation. We therefore stimulated cells in the treated group and the control group with 5 mM barium with no calcium in the buffer solutions. Event frequency per cell and cumulative number of events per cell are shown in Fig. S3(a) and (b). The event frequencies in the control group did not drop down but remained at a relatively constant level during the entire recording as previously reported (Haynes *et al.* 2007). Moreover, there was no significant difference between the event frequencies in the citalopram‐treated cells and the control cells, supporting the hypothesis that citalopram selectively increases the RRP. The amperometric spike parameters also show no difference between the two groups (Fig. S3c and d).

**Figure 4:**
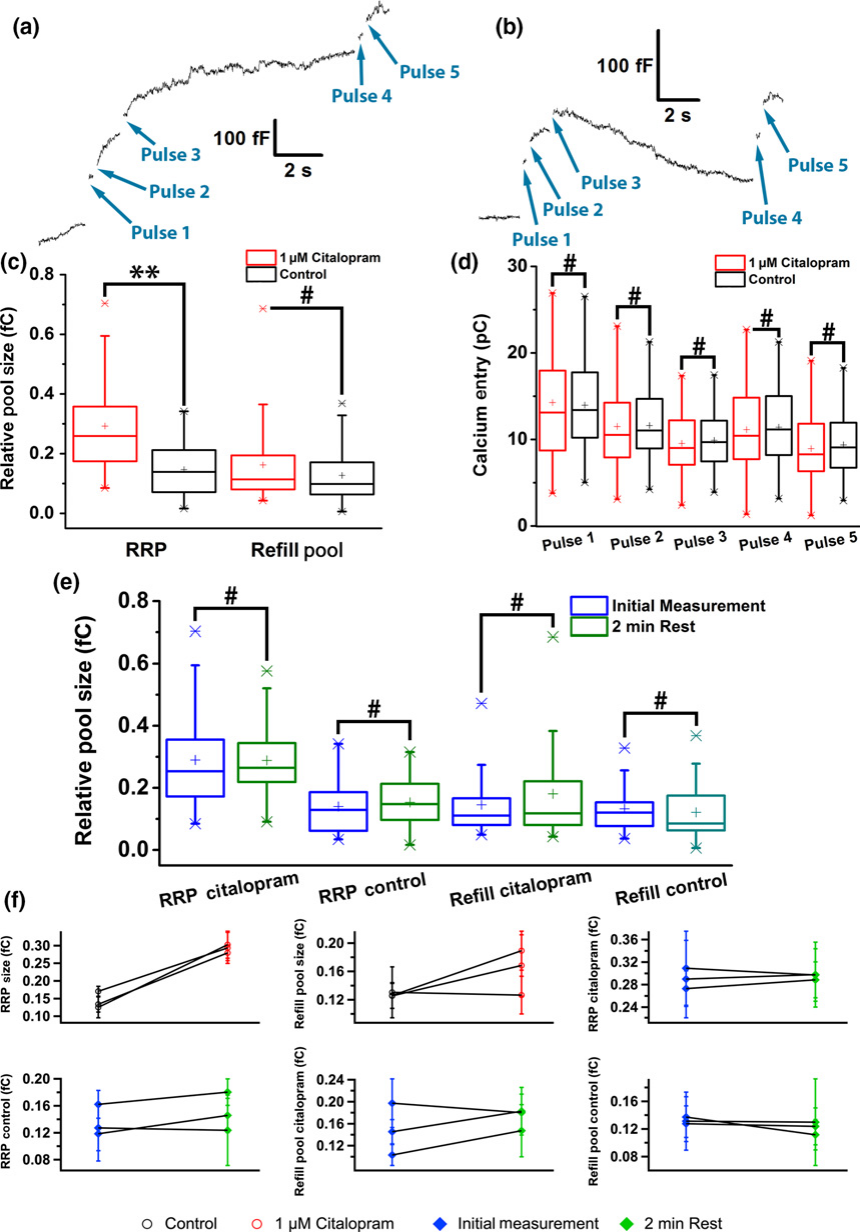
(a) Example patch‐clamp traces for a cell treated with citalopram and (b) a cell in the control group. (c) RRP and refill pool sizes, and (d) Average calcium current integrals for the five individual pulses in box plots. The citalopram‐treated group shows twice as large RRP size compared with the control group, but no difference on the refill pool size. The calcium entry levels were very close for all pulses. (e) RRPs and refill pool sizes of the citalopram‐treated group and the control group for the initial measurement and the second measurement after 2 min showed no significant differences. (f) Scatter plots for all pool sizes. Each data point represents the mean of the pool sizes of all cells from the same preparation. **p < 0.01, ^#^ *p *> 0.05. For control group, *n* = 24 cells, for citalopram‐treated group, *n* = 26 cells, 3 preparations.

To test the hypothesis that citalopram increases the RRP more directly, whole‐cell patch‐clamp capacitance measurements were performed using a double pulse protocol (Fig. 4a and b). The ratio between the capacitance changes ∆C_1_ and ∆C_2_ induced by the two subsequent pulses can be used to quantify the size of the exocytotic response and the size of the RRP. If the first pulse releases a fraction *p* of the RRP and the second pulse is assumed to release a similar fraction *p* of the remaining fraction, then the RRP pool size can be calculated as $RRP=\frac{\Delta C_{1}+\Delta C_{2}}{1-{\left(\frac{\Delta C_{2}}{\Delta C_{1}}\right)}^{2}}$ (Gillis *et al.* 1996). The statistical analysis (Fig. 4c) showed that indeed the RRP size of the drug‐treated group was about twice as large as that of the control group. Figure 4d shows that the calcium entry was unchanged demonstrating that the increased RRP in citalopram‐treated cells was not because of a change in calcium entry. However, the pool sizes obtained after ~ 10 s waiting time following the first two pulses for refilling were similar for both the groups and only slightly smaller than the initial RRP size of the control group, indicating that the extra priming effect of citalopram takes longer than the RRP recovery in untreated cells. Figure 4e shows the comparison of RRPs and refill pool sizes of the two groups between the first measurement and a second measurement 2 min after the first one (pulses 4,5). After 2 min rest, the RRPs and the refill pool sizes were restored to the initial values also for the citalopram‐treated cells, indicating that the enhanced priming produced by citalopram treatment occurs on the minute time scale, that is, slower than the pool refilling in control cells. In contrast to citalopram, bupropion does not change the RRP size (Fig. S4d and e).

## Discussion

Amperometry is one of the most commonly used methods to characterize quantal release events. Each exocytosis event corresponds to an amperometric spike in the recording, and the spike provides precise details about the released neurotransmitters in a single‐release event. However, the properties of the spikes vary from cell to cell (Colliver *et al.* 2000a) and therefore a large number of quantal release events from many cells must be measured and analyzed to achieve statistical significance in order to quantify the effects of drugs on transmitter release. The general statistical analysis for amperometry determines the median values for each spike parameter for each cell and then takes the averages from all cells such that the ‘n’ for the statistical tests is not the number of spikes but the number of cells. The conventional way of using a carbon fiber electrode for amperometry measures one cell per time, and therefore requires labor‐intensive efforts to obtain a sufficient number of recordings. CMOS‐based integrated circuit biosensors designed for amperometry measurement with appropriate surface microwell structures are beneficial replacements for the carbon fiber electrode method, reducing the time consuming process from months or weeks to days (Ayers *et al.* 2007; Kim *et al.* 2013; Huang *et al.* 2018).

Bupropion is recognized as a dopamine (DA) and norepinephrine (NE) reuptake inhibitor (Ferris and Beaman 1983; Ascher *et al.* 1995; Damaj *et al.* 2004). It acts, however, also as a vesicular monoamine transport enhancer which promotes the uptake of cytoplasmic DA into the secretory vesicles (Rau *et al.* 2005). This may explain the large increase in the average amount of catecholamine released from individual vesicles (Fig. 2e and f) because more DA may be accumulated inside the vesicles. Alternatively, bupropion may increase the fraction of transmitter that is released from an individual vesicle. It is expected that with increased accumulation of neurotransmitters in the vesicles the vesicle volume and membrane area of the vesicle will increase (Bruns *et al.* 2000; Colliver *et al.* 2000b). In whole‐cell patch‐clamp capacitance measurements, which quantify the amount of vesicle membrane incorporated into the plasma membrane by exocytotic vesicle fusion in response to pulse stimulation, the capacitance changes in bupropion‐treated cells were unchanged compared to control cells (Fig. S4a–c). Together with the unchanged release frequencies (Fig. 2c and d), this result could be taken as evidence that there was no increase in the size and membrane area of the vesicles in the bupropion‐treated cells. However, it is possible that bupropion was not effective during the patch‐clamp experiments because it might be diluted by the pipette solution or it may require some cytoplasmic components that were washed out during the measurement. Another possibility is that bupropion may modulate fusion pore expansion and shift the fusion mode from partial release to full release in the amperometry experiments, leading to the potentiation of quantal size, maximum current, and foot amplitude (Fig. 2e and f). An increase in the fraction of molecules released in a single event without a change in vesicular contents was previously observed for the synaptobrevin 2 W89A/W90A mutant, myosin II and actin‐directed compounds, and the dynamin‐1 knock‐out in chromaffin cells (Doreian *et al.* 2008; Fang *et al.* 2013; Wen *et al.* 2016; Wu *et al.* 2018).

Up to 50% of PD patients are suffering from depression in addition to motor dysfunction (Grimes *et al.* 2012; Cooney and Stacy 2016). It has been proposed that bupropion may be used as treatment for PD patients with depression because of its function as a reuptake inhibitor for both DA and NE without the side effects associated with serotonin (Raskin and Durst 2010). Clinical studies also reveal that bupropion improve both depression and motor symptoms in some patients (Goetz *et al.* 1984; Cooney and Stacy 2016). However, the reported cases and trials are few in numbers (Cooney and Stacy 2016) and the efficacy of the drug for PD is not conclusive. Here, we demonstrate that bupropion significantly increases the quantal size of individual transmitter release events, in an amount very similar to l‐Dopa (Pothos *et al.* 1996; Gong *et al.* 2003), which may explain its beneficial effects for the treatment of both depression and motor deficiencies in PD patients.

Citalopram is a selective serotonin reuptake inhibitor (SSRI) and is not expected to induce a significant change in quantal size and other parameters of single release events consistent with the results of Figure 3(c) and (d). However, the release frequency for the first 90 s was boosted compared with the control group (Fig. 3a and b), which we have shown to reflect an increase in the RRP size by patch‐clamp capacitance measurements (Fig. 4c). The ~ 3‐fold increase of the release frequency during the first 90 s of sustained high [K^+^] stimulation exceeds the ~ 2‐fold increase of RRP size measured with a 400 ms double pulse stimulus. While the refilling of the RRP 10 s after its depletion was not different from that in control cells, the RRP was again robustly increased after 2 min (Fig. 4e), indicating the RRP increase promoted by citalopram is a slower process that occurs on the time scale of minutes and therefore the continued potentiation of vesicle priming on the minute time scale contributes to the enhanced release in the 90 s long amperometric recordings.

In AD, synaptic transmission is markedly reduced but the details are highly complex (Ovsepian *et al.* 2018). FDA‐approved drugs for AD can slow down the worsening of the AD symptoms, such as memory deficit, but only with small benefits (Adlimoghaddam *et al.* 2018). Recent studies showed that citalopram restores short‐term memory and treats depression‐like behavior in APPswe/PSEN1dE9 mice, preventing the advance of AD‐like pathology by increasing the number of neurons in the cortex with active parvalbumin (Zhang *et al.* 2018), a calcium‐binding protein modulating synaptic activities (Caillard *et al.* 2000). Changes in transmitter release are closely associated with AD neuropathology. The cognitive dysfunction in AD is associated with defects in presynaptic vesicle proteins, such as a reduction of synaptobrevin in AD brain, which results in a loss of neurotransmitter release (Sze *et al.* 2000). Sufficient levels of neurotransmission are critical for synaptic maintenance and prevention of AD (Keating *et al.* 2008). We show here that citalopram increases the readily releasable pool size, which leads to increased release frequency and thus enhances neurotransmitter release. The RRP increase by citalopram may explain its efficacy in restoring memory deficit in AD patients (Harmer *et al.* 2002; Zhang *et al.* 2018), making it a potentially effective treatment for both AD symptoms and depression. The mechanism by which citalopram increases the RRP still needs to be elucidated. It is likely not a consequence of its canonic function as an SSRI. However, citalopram has additional targets and inhibits platelet activation independent of SERT, possibly via inhibition of Rap1 activation (Roweth *et al.* 2018). Interestingly, it has been reported that Rap1 deletion increases exocytosis in mouse cortical neurons, possibly by regulating calcium channel expression (Subramanian *et al.* 2013). However, further work will be needed to determine the detailed pathway by which citalopram enhances the RRP.

It is worth noting that the catecholamine release from neuroendocrine cells differs from synaptic transmissions in some ways, such as the active zones and protein rich areas (Zhai and Bellen 2004). Nevertheless, this study is focused on the effect of drugs on the quantal size, release frequency and kinetics of vesicle release event. While chromaffin cells are not neurons, the vesicle fusion, docking, and priming mechanisms, and the proteins associated with the release from RRP are identical or very similar between the two types of cells (Burgoyne and Morgan 1998; Stevens *et al.* 2011). Therefore, chromaffin cells are a valuable model to advance our understanding of the mechanisms of transmitter release and their modulation. The ease of preparing and culturing chromaffin cells on the CMOS chip also makes it a powerful model for high‐throughput drug screening. In future experiments, the human neuroblastoma cell line SH‐SY5Y may be used as a neuronal model for dopamine release. These cells have recently been used successfully for amperometric recording using a similar CMOS chip (White *et al.* 2018). The CMOS chips used in our study are not designed for measurement of glutamate. However, with the recent development of fast responding glutamate sensors (Wang *et al.* 2019), the CMOS chip technology may in the future also be applicable to directly perform testing of drugs with respect to modulation of glutamate release.

Modulation of exocytosis is a key drug target and affected by molecular manipulations (Sulzer and Pothos 2000). In this work, we showed that high throughput single cell amperometry using a CMOS device with incorporated microwells revealed a dramatic increase in the initial release frequency by citalopram. This result suggested an increase in the RRP, which we confirmed and characterized by follow‐up patch‐clamp capacitance measurements. Bupropion increases the amount of transmitter release from individual vesicles. Both drugs may thus be candidates for treatments of neurological disorders where transmitter release is impaired.

## Supporting information


**Figure S1.** Box analysis of the amperometry parameters for the bupropion‐treated cells.
**Figure S2.** Distribution analysis of the quantal size of bupropion‐treated cells.
**Figure S3.** Barium stimulation experiment for the citalopram‐treated group and the control group.
**Figure S4.** Patch‐clamp recordings for the bupropion‐treated cells.
**Table S1.** *P* values for all parameters used for significance determination.
