# Supplementary material for: Drug testing complementary metal‐oxide‐semiconductor chip reveals drug modulation of transmitter release for potential therapeutic applications
Source: J Neurochem. 2019 Jul 31;151(1):38–49. doi: 10.1111/jnc.14815 (PMC6837173; doi:10.1111/jnc.14815)
Supplement: Supplementary file 1 — Figure S1. Box analysis of the amperometry parameters for the bupropion‐treated cells. Figure S2. Distribution analysis of the quantal size of bupropion‐treated cells. Figure S3. Barium stimulation experiment for the citalopram‐treated group and the control group. Figure S4. Patch‐clamp recordings for the bupropion‐treated cells. Table S1. P values for all parameters used for significance determination. [file JNC-151-38-s001.pdf]

Drug Testing CMOS Chip Reveals Drug Modulation of Transmitter Release for Potential Therapeutic Applications

Meng Huang,[*,†] Shailendra S. Rathore,[†] Manfred Lindau[†]

[*]*Department of Materials Science and Engineering, Cornell University, Ithaca, NY, USA*

[†]*School of Applied and Engineering Physics, Cornell University, Ithaca, NY, USA*

Supplementary Information

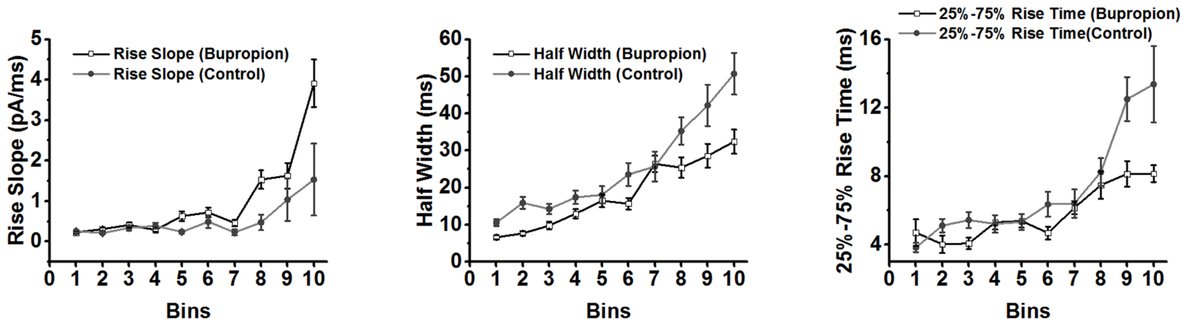

Quantal Size Bin Distribution										
Bins	1	2	3	4	5	6	7	8	9	10
Start (pC)	0.0023	0.019	0.030	0.040	0.053	0.074	0.099	0.14	0.20	0.37
End (pC)	0.019	0.030	0.040	0.053	0.074	0.099	0.14	0.20	0.37	6.8

Figure S1. Bupropion preferentially enhances the kinetics of larger vesicles. All amperometry events were sorted by ascending order and divided into 10 bins, regardless of treatment with bupropion or not. Each bin contained the same number of events and afterwards events of the bupropion treated group and the control group were analyzed separately to extract their kinetic parameters, including the rise slope of the amperometry spike, the half width of the spike and the 25%-75% rise time of the spike. The 10th bin (0.37 pC-6.8 pC) may include spikes that have much higher quantal sizes than others, resulting in a shift in these parameters as larger vesicles will generally have slower kinetics. However, the average quantal sizes of the 10th bin for the bupropion treated group and the control group were 0.81 ± 0.08 pC and 0.86 ± 0.19 pC, showing that the extra-large vesicles were fewer in number and would not significantly affect the final results . It was found that bupropion speeded up the release kinetics with higher rise slope and smaller half width for vesicles larger than 0.14pC (starting from box or interval 8) The 25%-75% rise time also deviated between the two groups, but only for vesicles larger than 0.20 pC (starting from box or interval 9), indicating a slower rising phase for the control group. n is total number of individual events analyzed. n = 1239 amperometric spikes from 43 control cells and 49 bupropion treated cells, 4 preparations.

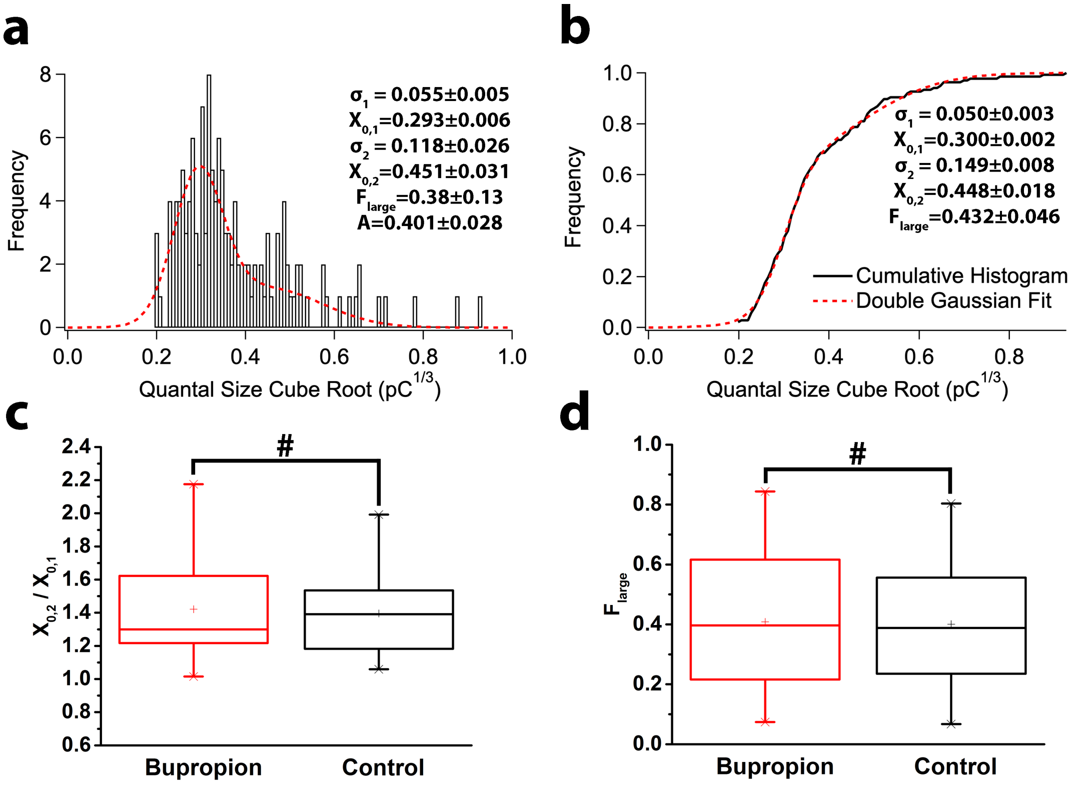

Figure S2. Distribution analysis of the quantal size of bupropion treated cells. The quantal sizes of all events were taken the cubic root for analysis. (a) The histogram of the cube root quantal size of a cell with 113 events. The red dashed line shows the double gaussian fit with equation $y = A(\frac{1-F_{large}}{\sigma_1} e^{-\frac{(x-X_{0,1})^2}{2\sigma_1{}^2}} + \frac{F_{large}}{\sigma_2} e^{-\frac{(x-X_{0,2})^2}{2\sigma_2{}^2}})$. (b) The cumulative histogram of the cube root quantal size of the same cell in (a) with the frequency normalized to one. The red dashed line shows the double cumulative gaussian fit with equation $y = \left(1 - F_{large}\right) \frac{1+\mathrm{erf}\left(\frac{(x-X_{0,1})^2}{2\sigma_1{}^2}\right)}{2} + F_{large} \frac{1+\mathrm{erf}\,(\frac{(x-X_{0,2})^2}{2\sigma_2{}^2})}{2}$. The two double gaussian fits in (a) and (b) show reasonable fit to the original data and close parameters. The cumulative gaussian fit was used for all other cells as the errors were much lower. (c) The ratio of the means of the double gaussians for the bupropion treated and the control groups. No significant difference was observed. (d) The ratio of the larger vesicle group, A_{large}, show no difference between the bupropion treated group and the control group. $^{\#}P$>0.05. For control group, n = 35 cells, for bupropion treated group, n = 38 cells, 4 preparations.

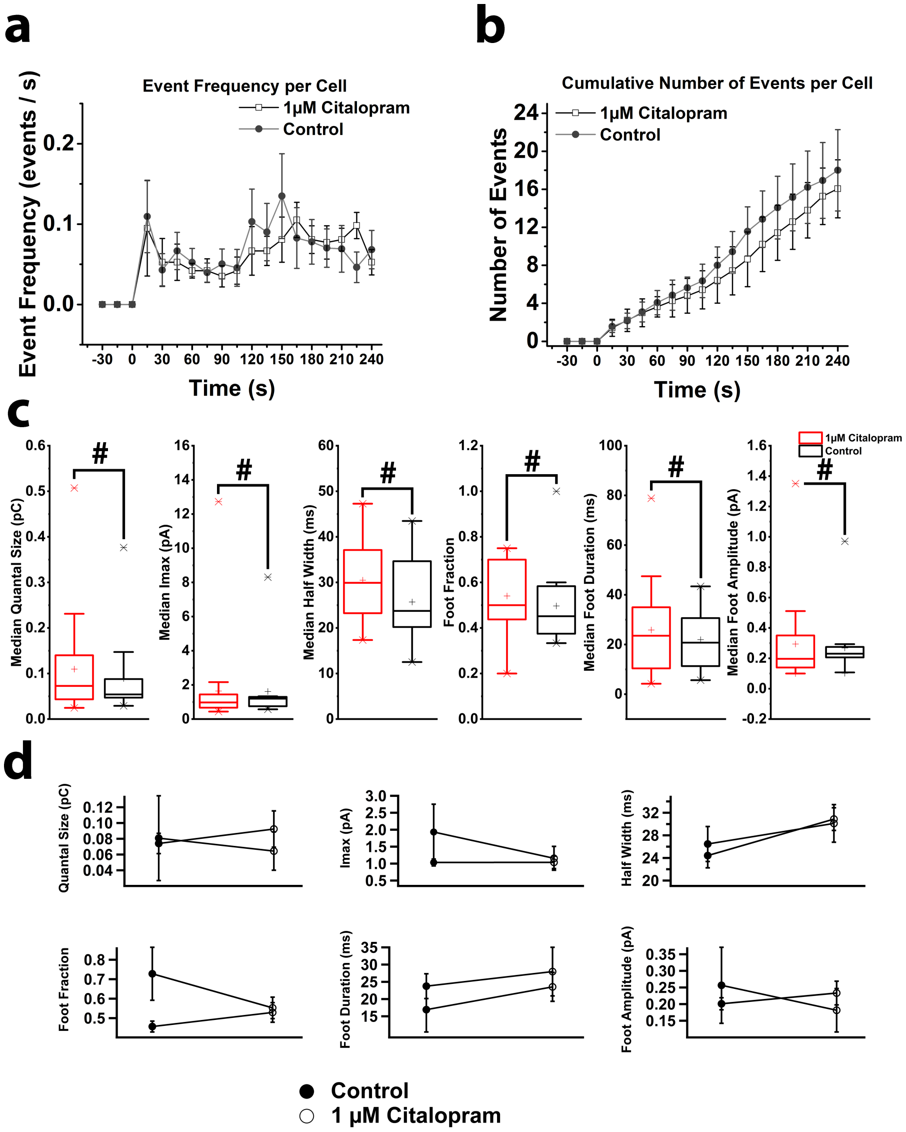

Figure S3. Citalopram treated group and the control group were stimulated with 5 mM barium with no calcium in the buffer solutions. (a) Average event frequency per cell during the measurement and (b) cumulative number of events per cell during the entire recording. (c) Box plots of median amperometry parameters for cell treated with 1 μM citalopram (red) and the control group (black). No significant differences were observed. *P* values for the quantal size, maximum current, half width, foot fraction, foot duration and foot amplitude are 0.3625, 0.56, 0.126, 0.1777, 0.743 and 0.9709, respectively. (d) Scatter plots for all amperometry parameters. Each data point represents the mean of medians of all cells from the same preparation. All error bars are in SEM. $^{\#}P>0.05$. For control group, n = 15 cells, for citalopram treated group, n = 19 cells, 2 preparations.

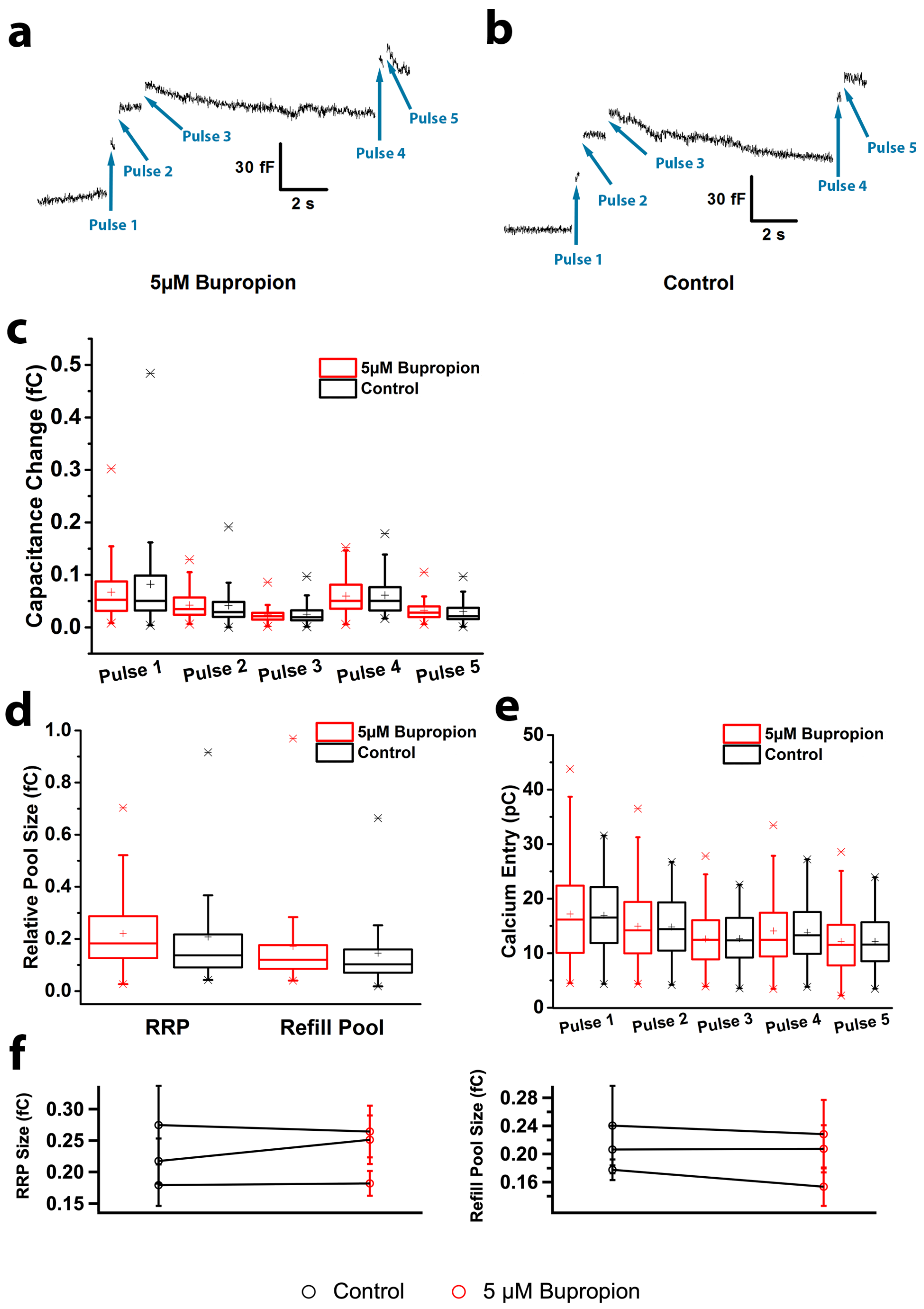

Figure S4. (a) Example patch clamp recordings for a bupropion treated cell and (b) a cell in the control group. Blue arrows indicate the positions of the five pulses. (c) Capacitance changes ΔC_M, (d) RRP and refill pool sizes and (e) Average calcium entries for the five individual pulses in box plots. (f) Scatter plots for pool sizes. Each data point represents the mean of the pool sizes of all cells from the same preparation. No significant difference was observed for ΔC_M, RRP size, refill pool size and calcium entries. For control group, n = 35 cells, for bupropion treated group, n = 38 cells, 3 preparations.

Group	Subgroup	Parameter	P value
Bupropion Amperometry		Quantal Size	**0.0003**
Bupropion Amperometry		Quantal Size Cubic Root	**0.0001**
Bupropion Amperometry		Peak Current	**0.0014**
Bupropion Amperometry		Half Width	0.7277
Bupropion Amperometry		Foot Fraction	0.1310
Bupropion Amperometry		Foot Duration	0.7159
Bupropion Amperometry		Foot Amplitude	**0.0447**
Citalopram Amperometry		Quantal Size	0.4196
Citalopram Amperometry		Quantal Size Cubic Root	0.4725
Citalopram Amperometry		Peak Current	0.1509
Citalopram Amperometry		Half Width	0.4684
Citalopram Amperometry		Foot Fraction	0.2798
Citalopram Amperometry		Foot Duration	0.5027
Citalopram Amperometry		Foot Amplitude	0.0537
Patch Clamp Citalopram		RRP	**0.0002**
Patch Clamp Citalopram		Refill Pool	0.2630
Patch Clamp Citalopram	Calcium Entry	Pulse 1	0.3947
Patch Clamp Citalopram	Calcium Entry	Pulse 2	0.5110
Patch Clamp Citalopram	Calcium Entry	Pulse 3	0.8574
Patch Clamp Citalopram	Calcium Entry	Pulse 4	0.7205
Patch Clamp Citalopram	Calcium Entry	Pulse 5	0.4068
Patch Clamp Citalopram	Initial Pulses and Pulses after 2 min	RRP Citalopram	0.5395
Patch Clamp Citalopram	Initial Pulses and Pulses after 2 min	RRP Control	0.6396
Patch Clamp Citalopram	Initial Pulses and Pulses after 2 min	Refill Citalopram	0.6506
Patch Clamp Citalopram	Initial Pulses and Pulses after 2 min	Refill Control	0.4963
Bupropion Quantal Size Distribution Analysis		$X_{0,2} / X_{0,1}$	0.7428
Bupropion Quantal Size Distribution Analysis		A_{large}	0.8976
Patch Clamp Bupropion		RRP	0.0827
Patch Clamp Bupropion		Refill Pool	0.1370
Patch Clamp Bupropion	Capacitance Changes	Pulse 1	0.7277
Patch Clamp Bupropion	Capacitance Changes	Pulse 2	0.2748
Patch Clamp Bupropion	Capacitance Changes	Pulse 3	0.6153
Patch Clamp Bupropion	Capacitance Changes	Pulse 4	0.9181
Patch Clamp Bupropion	Capacitance Changes	Pulse 5	0.1624
Patch Clamp Bupropion	Calcium Entry	Pulse 1	0.8100
Patch Clamp Bupropion	Calcium Entry	Pulse 2	0.8016
Patch Clamp Bupropion	Calcium Entry	Pulse 3	0.8128
Patch Clamp Bupropion	Calcium Entry	Pulse 4	0.7961
Patch Clamp Bupropion	Calcium Entry	Pulse 5	0.7490

Table S1. P values for all parameters used for significance determination. P values in bold are less than 0.05, indicating significant differences for the corresponding parameters.
